# Performing Percutaneous Dilational Tracheostomy without using Fiberoptic Bronchoscope

**Published:** 2020-01

**Authors:** Siamak Yaghoubi, Nilofar Massoudi, Mohammad Fathi, Navid Nooraei, Marzieh Beygom Khezri, Sareh Abdollahi

**Affiliations:** 1 Department of Anaesthesiology, Qazvin University of Medical Sciences, Qazvin, Iran,; 2 Clinical Research and Development Unit at Shahid Modarres Hospital, Department of Anaesthesiology, Shahid Beheshti University of Medical Sciences, Tehran, Iran.,; 3 Critical Care Quality Improvement Research Center, Shahid Beheshti University of Medical Sciences, Tehran, Iran.,; 4 Clinical Research and Development Unit at Shahid Modarres Hospital, Department of Anaesthesiology, Shahid Beheshti University of Medical Sciences, Tehran, Iran.

**Keywords:** Percutaneous dilatation tracheostomy, fiberoptic bronchoscopic guidance, Outcomes

## Abstract

**Background:**

Percutaneous tracheostomy is an elective method that is increasingly being taken up in the intensive care unit alongside the patient’s bed. In many centers, bronchoscopy is used, but the necessity of using bronchoscopy in percutaneous tracheostomy has not yet been determined. Discontinuing use of bronchoscopy can potentially reduce the cost and increase the efficiency of percutaneous tracheostomy. Therefore, in this study, we performed a percutaneous dilatational tracheostomy without using fiberoptic bronchoscopy.

**Materials and Methods:**

This study was performed as a descriptive epidemiological survey among 70 patients in Shahid Rajaei Hospital of Qazvin in 2015 and 2016. The results were assessed in the patients.

**Results:**

In this study, pneumothorax, trauma, major and minor bleeding, cuff leak and change to surgical procedures as well as accidental extubation were not seen. However, subcutaneous emphysema, mal-position and hypoxia each were seen in one patient (1.4%).

**Conclusion:**

Totally the results demonstrated that percutaneous dilatation tracheostomy without fiberoptic bronchoscopic guidance is useful and safe.

## INTRODUCTION

Tracheostomy is one of airway management options in patients who need long term ventilation and airway protection (1).

Percutaneous (dilatational) tracheostomy (PDT) was first presented by Sheldon in 19571 and years later added as a substitute to surgical tracheostomy (ST) in intensive care units (ICUs). With the daily advancement of this technique (PDT) and the use of advanced tools, this technique is gradually performed as a standard open surgery technique in the operating room (2).

Tracheostomy consists of opening the trachea from anterior cervical region and communicating tracheal space to outside by a plastic or metal cannula. It is a common surgical procedure especially in traumatic patients and those who are under mechanical ventilation for a long time. It is usually done in two ways; Percutaneous Dilational Tracheostomy (PDT) and Surgical Tracheostomy (3, 4). Although PDT method is faster and easier, it needs to be converted to open surgery in 7 percent of cases (3). The physician experience should not be neglected that clearly reduces complications of either method (5). Although other factors like, age, gender, and history of previous intubation, have no effect on incidence of complications or successful PDT placement (6), differences in surgical methods and dealing with the anatomical problems can have a significant impact on PDT outcomes and reduction of its complications (3, 4).

In old traditional way, patient was transferred from intensive care unit (ICU) to operating room, and presence of surgery team performed the tracheostomy (7). In percutaneous tracheostomy method, surgery is done by ICU physician beside the patient’s bed with no need for operating room. In this way there will be less tissue damage, bleeding risk and infection due to limited tissue incision (5). Also bronchoscopy is not obligatory in performing PDT (6, 8). Recent studies in trauma patients showed that using bronchoscopy does not change complications of tracheostomy (9). There is no consensus about using bronchoscope during PDT as routine or in special conditions, and in some centers bronchoscopy is never used during PDT (6, 10). Whereas some studies recommend using bronchoscope as a guide during PDT, and mention that its usage, reduces complications like pneumothorax, and tracheal posterior wall damage, and is useful to treat intrabronchial hemorrhage (9). Using bronchoscope increases the safety of PDT procedure, but can cause hypoventilation, hypercarbia, and respiratory acidosis. Also, it increases the cost of percutaneous tracheostomy and will necessitate the presence of another specialist because of the complexity of the procedure (11, 12). Therefore, in this study we decided to evaluate the complications of PDT without using bronchoscope.

## MATERIALS AND METHODS

This study was done as a descriptive epidemiological survey among 70 patients who were admitted in ICU and candidate for tracheostomy in Shahid Rajaei Hospital of Qazvin in 2014 and outcome of percutaneous dilational tracheostomy without using fiberoptic bronchoscope was investigated. This study had the Ethic code of: “IR. QUMS.REC. 1396.140” from Ethics committee of Research Department of Qazvin University of Medical Sciences and registered in IRCT with code of:”RCT2017061923473N3”.

All cases of PDT were performed at patient’s bedside and in ICU. Firstly, patients underwent general anesthesia with 0.01 mg/kg midazolam, 2 mcg/kg fentanyl, 1–2 mg/kg propofol and 0.5 mg/kg atracurium under complete cardiac and respiratory monitoring. Then patient neck was positioned at hyperextension by using sub shoulder roll and target site was cleaned with 2% chlorhexidine alcohol. Blood pressure, heart rate and rhythm and SPO_2_ were continuously monitored during procedure. Patients were under mechanical ventilation with FIO_2_ 100% and respiratory rate of 12 and 8 cc/kg tidal volume. At the beginning anatomy was determined precisely, by marking the region 2 to 3 centimeters above sternal notch between cricoid and sternal notch, and to reduce bleeding, 60 mg lidocaine with 1/10000 epinephrine was injected subcutaneously at the specified region. Then a 1.5 cm horizontal incision was given by scalpel, and pretracheal muscles were exposed. With accurate palpation of thyroid cartilage, tracheal rings were revealed, and between 2nd and 3rd midline cartilage (connected to a 10 cc syringe prefilled with 2 cc of distilled water) trachea was entered with a 14 gauge needle. Correct insertion of needle was confirmed by aspirating air. Then the syringe was removed and metal catheter was passed through the needle and inserted into trachea for about 10 cm, and free movement of guide wire inward or outward was checked. Thereafter, the needle was withdrawn and a 14-french plastic dilator was passed over the guide wire. While the guide wire was still in trachea, the dilator was taken out and next white long dilator passed over the guide wire, and Rhino dilator passed over the white dilator into trachea up to the skin level line. Afterwards rhino dilator was removed and through the metal guide wire and white dilator, appropriate tracheostomy tube was passed and placed into the trachea. Then the metal guide wire and white dilator were removed. After symmetrical bilateral auscultation of both lungs and confirmation of correct tracheostomy tube placement, orotracheal or nasotracheal tube was removed, and tracheostomy tube fixed by special banding. Portable Chest x-ray was obtained from all patients, one hour after procedure. Amount of blood loss during procedure, SPO_2_ level below 90%, accidental extubation, tracheal tube cuff rupture and posterior tracheal wall rupture were recorded. Patients were followed up for incidence of pneumothorax, subcutaneous emphysema, incorrect positioning of tracheostomy tube and also tracheostomy associated death up to 24 hours after procedure.

## RESULTS

In this study, 70 ICU patients underwent PDT surgery, and were monitored for incidence of complications. The descriptive results are presented in this segment.

As it is shown in table 1, 52 (74.3%) patients were males. Average age was 46.9 years and average duration of hospitalization in ICU was 21.8 days.

**Table 1.** Frequency distribution of demographic characteristics

**Variable**	**Average ± SD**	**Range**
**Age (years)**	46.9 ± 3.17	19 – 85
**Duration of ICU admission (days)**	21.8 ± 6.7	9 – 42
**Gender**	Male	Female
	52 (74.3%)	18 (25.7)

As it is shown in table 2, pneumothorax, trauma to posterior tracheal wall, minor or major hemorrhage, tracheal tube cuff rupture and death 24 hours after tracheostomy and changing procedure to surgical tracheostomy and accidental extubation during surgery were never witnessed among any of the patients. Subcutaneous emphysema, incorrect positioning of tracheostomy tube and hypoxemia or decrease in SPO_2_ during surgery were seen in 1 patient (1.4%).

**Table 2.** Frequency distribution of complications

**Complications**	**Frequency**	**Percentage**
**Pneumothorax**	0	0
**Trauma to posterior tracheal wall**	0	0
**Minor bleeding (less than 10 cc)**	0	0
**Major bleeding (need for transfusion)**	3	4.2
**Tracheal tube cuff Rupture**	4	5.7
**Death during 24 hours of tracheostomy**	0	0
**Changing the procedure to tracheostomy surgery**	0	0
**Subcutaneous emphysema**	1	1.4
**Misplacement of tracheostomy tube**	1	1.4
**Accidental extubation during the procedure**	0	0
**Hypoxia and desaturation during the procedure**	1	1.4

## DISCUSSION

In this study we performed PDT without fiberoptic bronchoscopy for 70 patients that could not be extubated at least 2 weeks after intubation; and complications during and 24 hours after procedure were also investigated. The results showed that pneumothorax and trauma to posterior tracheal wall and minor or major bleeding and tracheal tube cuff rupture and death during 24 hours after tracheostomy and changing procedure to surgical tracheostomy and accidental extubation during the procedure were not seen in any of the patients. Subcutaneous emphysema, tracheal tube misplacement and mild hypoxia during procedure were seen in only 1 patient (1.4%).

In a study that was done in from 2007 to 2016 on 649 ICU admitted trauma patients who were candidate for tracheostomy, 289 patients underwent PDT with bronchoscope and 360 patients had undergone PDT without bronchoscope. There was no significant difference in complications between the two groups. In this study it was mentioned that using bronchoscopy is beneficial theoretically (12). In a retrospective study in 3162 ICU admitted trauma patients PDT surgery without bronchoscope was done in years 2001 to 2011 and outcomes were analyzed. In this study, the procedure was successful in 99.62 patients including extreme obese patients. Major airway complications occurred in 12 patients (0.38%), 5 of whom died (0.16%). 3 of them needed open surgery because of loss of airway patency (0.09%) and 1 (0.03%) for airway bleeding. Two deaths happened due to airway obstruction (0.06%) (13). In another study, 80 patients who were connected to ventilator in ICU, underwent PDT without bronchoscopy (with Rhino technique). This study showed 11.6 % of patients had complications like tracheal tube cuff rupture; failure to do the procedure, losing the air way, bronchospasm, subcutaneous emphysema, drop or rise of blood pressure and 9.1% had early complications after surgery. Rupture of tracheal tube cuff and self-limiting hemorrhage from the site of tracheostomy occurred in 2 and 3 patients, respectively. None of the complications led to death of patients (14). Since patients in this study were admitted because of various reasons, the results could not be adjusted to our study.

Fiberoptic bronchoscope is widely used in ICU to reduce complications, but there are plenty of evidences about complications during bronchoscopy (15). In a study which was done on 243 trauma patients underwent PDT, fiberoptic bronchoscope was used in 32%, and complications were investigated. Sixteen complications happened; 11 in non-fiberoptic group, and 5 in fiberoptic group. Amount of bleeding in fiberoptic group was 3%, and 4% in non fiberoptic group. In addition 1.2% of cases in bronchoscope group had major complication of losing air way and cardiac arrest. This study concluded that routine use of fiberoptic bronchoscope is unnecessary, but can be useful in obese patients or those who have abnormal cervical anatomy (15). In another retrospective study in 2013 in trauma patients, it was concluded that fiberoptic bronchoscope is a very reliable method even in obese patients, but its routine use is not recommended (13).

Some studies recommend using fiberoptic bronchoscope as a guide during PDT and mention that it reduces complications like pneumothorax and posterior tracheal wall injury, and is useful to treat complications like intra bronchial hemorrhage (9).

There was no case of accidental extubation in our study. In a study which was done on 300 patients who were candidate for PDT without bronchoscopic guide, 6 (2%) accidental extubation, and 12 (4%) tracheal tube cuff rupture was reported, which happened in first 6 months of study and mostly because of assistant inexperience (16).

In Delaney et al. study, it was reported that number of bleeding, major complications and mortality in patients who had undergone PDT had no difference with those who had undergone open tracheostomy (17); point consistent with our study in which those complications never happened.

In our study, the amount of bleeding during surgery was minor and compatible with tracheostomy procedure and none of patients had major bleeding. Some studies have had paradoxical results.

In a review article which was done to investigate PDT fatal complications, 71 studies were analyzed. This study declared that the incidence of fatal complications of PDT is 0.17%, 31% of which occurred during procedure and 49.2% occurred during the next 7 days. Most causes of death included: hemorrhage (38%), air way problems (29.6%), tracheal perforation (15.5%), and pneumothorax (5.6%). In this study only 1 case of death following PDT happened (among 600 patients) (18). In another study which was done on 149 brain damaged patients who were candidate for PDT, amount of major bleeding in either (using or not using bronchoscope) was reported zero, which is consistent with our study results (15). In another study which was done on 104 ICU patients, who were admitted because of pneumonia (24%), sepsis (18.3%) and drug toxicity (6.7%) no case of major bleeding was witnessed; the reason could be less tissue damage and the tamponade effect of proper sized tracheostomy tube (19). It seems that the dissection used in this approach, leads to better visualization of blood vessels, and therefore a minor bleeding occurs, especially that cauterization for bleeding control is not used here (9).

In a meta-analysis study which consisted of 15 interventional article and about a thousand patients, complications of PDT was compared with open surgery. It was concluded that percutaneous group significantly had less infection and scar formation compared with open surgery group, but minor and major hemorrhage, tracheostomy tube displacement and death showed no significant difference between the two groups (20). In another study, no case of displacement occurred among patients who had undergone PDT without bronchoscope (19). There was no case of PDT associated death in the present study.

There was no case of pneumothorax in this study. In a study which was done on 149 patients who were candidate for PDT (because of pneumonia, burning, chronic respiratory failure and trauma) in years 2007–2015, there was no case of pneumothorax, which is consistent with our study results (9).

In another study which was done on 60 brain damaged patients, there was no case of pneumothorax or subcutaneous emphysema in either bronchoscope group or the other group who underwent PDT, similar to our study (15).

Multiple tracheal punctures, excessively tracheal opening during surgery and also posterior tracheal wall damage by catheter or guide wire, are the causes of subcutaneous emphysema (21). On the other hand, using fenestrated tracheostomy tube when the fenestrated part would be completely or partially out of tracheal lumen, would allow air leakage to the para tracheal space (22). In this study the patient was observed closely and chest x-ray was done to rule out pneumothorax. So it seems that the most probable reason would be anterior or posterior tracheal damage.

In this study, there was a case of oxygen desaturation down to 88–89% during procedure (4.1%). Duration of procedure had no difference with other cases and there was no anatomical abnormality in the patient. Oxygen saturation was 96–98% with FIO_2_ 100% before the procedure, and patient was hemodynamically stable. Although PDT is a safe procedure (23), in some studies it is mentioned that duration of PDT with bronchoscopy is significantly increased, and is variable from 9 to 21 minutes according to the specialist’s experience (12). The time range in this study was between 5 to 7 minutes. In a retrospective study on 300 patients who were candidate for PDT without bronchoscopy, incidence of hypoxemia was zero (16).

Considering chest x-ray findings and widespread atelectasis in the patient, decreased respiratory reserve, is probably the cause of hypoxemia.

## CONCLUSION

The technique of PDT without bronchoscopic guide, along with some corrections like pulling back tracheal tube with fairly inflated cuff, monitoring of expiratory volume by ventilator and making sure that guide wire moves easily forward and backward in every step, is a reliable technique. There were no more complications in this study compared with the similar ones. According to previous studies, complications of this technique would decrease as the experience increases.

Altogether the results of this study showed that performing percutaneous dilational tracheostomy without fiberoptic bronchoscopy is a safe and effective method that can lower expenses of health system compared with other methods. So this procedure can be done without bronchoscopic guide in patients.
